# Interaction effect of oxytocin receptor (*OXTR*) rs53576 genotype and maternal postpartum depression on child behavioural problems

**DOI:** 10.1038/s41598-019-44175-6

**Published:** 2019-05-22

**Authors:** Damee Choi, Kenji J. Tsuchiya, Nori Takei

**Affiliations:** 1 Research Centre for Child Mental Development, Hamamatsu University School of Medicine, Hamamatsu, Japan; 2 Institute of Psychiatry, King’s College London, London, UK

**Keywords:** Psychology, Neuroscience

## Abstract

Previous studies have reported interaction effects of oxytocin receptor genotype (rs53576) and environmental factors on mental health in youth. However, the findings are mixed, especially regarding the type of allele (i.e., A vs. G), and it remains unanswered whether such an interaction presents at an early stage of development. Thus, using a unique longitudinal birth cohort sample in Japan (*n* = 568), we examined whether there was an effect of the interaction between the *OXTR* rs53576 genotype and maternal postpartum depression, as an environmental risk, on behavioural problems in children. Child behavioural problems (internalising and externalising problems) were ascertained using the Strengths and Difficulties Questionnaire when children were 6 years old. Maternal postpartum depression was measured using the Edinburgh Postnatal Depression Scale when children were at 2 months and 10 months of age. The results revealed a significant effect in the interaction between *OXTR* rs53576 genotype and maternal postpartum depression on externalising problems in children with AA genotype (*β* = 0.136, 95% CI 0.032 to 0.240), but not in those with GG/GA genotype. This indicates that an interaction of vulnerable genotypes (i.e., A allele of *OXTR* rs53576) with an environmental burden (i.e. maternal postpartum depression) may be one of the potential elements that predisposes the infant to developing behavioural problems early in life. Hence, special attention needs to be paid to children exposed to environmental risks such as maternal postpartum depression, to facilitate the provision of appropriate care.

## Introduction

Oxytocin is a peptide hormone that is involved in a wide range of human behaviours, and its role is well known in maternal behaviours such as parturition and lactation^1,2^. Recent studies have shown that oxytocin also plays an important role in social behaviours such as trust, empathy, prosocial behaviours, attachment, and social anxiety (reviewed in^3,4^). In addition, intranasal administration of oxytocin is postulated to be a potential treatment for mental disorders such as autism, social anxiety, schizophrenia, and borderline personality disorder (reviewed in^5^).

Another line of investigation has shown that variation in the oxytocin receptor (*OXTR*) gene is associated with individual differences in social behaviours. The human *OXTR* gene is located on chromosome 3p25, spans 17 kb and contains four exons and three introns^6^. A single nucleotide polymorphism (SNP) within intron 3 of the *OXTR* gene, rs53576, is a widely investigated SNP due to its association with individual differences in social behaviours^7–9^. Furthermore, it has been reported that *OXTR* rs53576 is related to variability of phenotypes in mental health (reviewed in^10^). In effect, the A allele of *OXTR* rs53576 has been reported to be linked to a high level of depressive symptoms^11^, suicide attempts^12^, and a low level of positive affect^13^.

Genetic factors are known to influence mental health through interacting environmental factors such as maternal depression and child maltreatment (reviewed in^14,15^). This gene × environmental interaction effect has been rooted in several psychological models such as the diathesis-stress and differential susceptibility models. In the diathesis-stress model, it is hypothesized that genetic vulnerabilities lead to mental disorders through an interaction with a stressful environment^16^. On the other hand, the susceptibility model posits that some genetic variation is associated with not only a stressful environment, but also a supportive environment^17^, extending the diathesis-stress model. This gene × environment interaction effect on mental health has also been reported for the *OXTR* rs53576 genotype. In a study by Thompson *et al*.^18^, for example, the A allele, but not the G allele, was demonstrated to be related to depressive symptoms in adolescents whose mother was afflicted with depression, which was, in fact, viewed as an environmental risk factor in the study. These studies suggest that oxytocin genotype, especially inheritance of the A allele, may play a role in susceptibility to disturbed mental health by interacting with adverse environments. However, findings are mixed. The G allele, by contrast, has been reported to be involved in the association between childhood maltreatment and subsequent mental health problems such as depression^19^, internalising problems^20^, and conduct problems^21^. Thus, inconsistent findings of the interaction effect of *OXTR* rs53576 genotype × environment on mental health in warrant further investigation.

As mentioned above, previous studies of *OXTR* rs53576 in relation to mental health, especially *OXTR* rs53576 genotype × environment interaction effects, have focused mainly on adolescents^18,21,22^ and young adults^19,23^. Given that child well-being is linked to adolescent and adult mental health (e.g.^24,25^) and early intervention in children with mental health problems has been advocated to promote optimal outcomes^26,27^, it would be of considerable value to explore this issue in populations of children. To our knowledge, only two studies have examined *OXTR* rs53576 genotype and environment interaction effects on mental health in children^28,29^. The previous studies did not demonstrate any significant interplaying effects. However, one study^28^ investigated the effect of interaction between environmental adversity (child maltreatment) and *OXTR* rs53576 genotype on coping functioning (strength)—that is, resilience in children in low-income families, especially in ethnic minority populations (African Americans). The other study^29^ considered the prenatal period as the risk period for environmental exposure; in that prenatal maternal stress, as opposed to childhood adverse experiences, was explored as an environmental risk potentially interacting with genotype. In addition, the latter study also focused on a rather specific phenotype, i.e., autistic traits, as a consequence of the influence. Although these negative results do not prompt further exploration, findings remain inconclusive since such conceptually narrow populations were investigated^28^ and the risk period for exposure and outcome phenotype were too specific^29^. The present study focused on maternal postpartum depression, since it has a negative impact on child mental health (e.g.^30,31^) and as many as 10% of mothers suffer from this condition^32^. This suggests that a large number of newborn babies are exposed to environmental risks during the first year of life, which is the most vulnerable developmental stage^30,33^. As a measure of mental health, we assessed internalising and externalising problems since these disturbances are strongly related with the interaction effect of *OXTR* rs53576 genotype × environment^21,22^ and have been reported to be predictors of mental health problems in adults, such as depression and anxiety disorders^24,25^. Furthermore, we utilized data from a longitudinal birth cohort of mother-child dyads. Prior studies have shown that the *OXTR* rs53576 A allele-specific genotype is associated with susceptibility to a disturbed mental state; thus, we hypothesized that the association, if any, between maternal postpartum depression and child behavioural problems, as represented by internalising problems and/or externalising problems, would be found in the A allele, but not the G allele, of *OXTR* rs53576. Herein, an attempt was made to investigate the interaction effect of *OXTR* rs53576 genotype × maternal depression on child behavioural problems, using a representative sample of the general population in Japan.

## Results

### *OXTR* genotyping

Genotyping of *OXTR* rs53576 revealed that 77 children had the GG genotype (13.6%), 257 children the GA genotype (45.3%), and 234 children had the AA genotype (41.2%) (Hardy-Weinberg equilibrium: χ^2^ = 0.232, *p* = 0.630). This genotypic frequency in the participants was consistent with those of previous studies which have reported that the frequency of the AA genotype of *OXTR* rs53576 is relatively high for populations in East Asia^7,22,34^. Given that patients with the GG and GA genotypes share common psychological characteristics^13,19^ as mentioned above, we amalgamated subjects with GG and GA genotypes into one group, as has been done in previous studies^13,19,22,34^.

### Child behavioural problems

**Figure 1:**
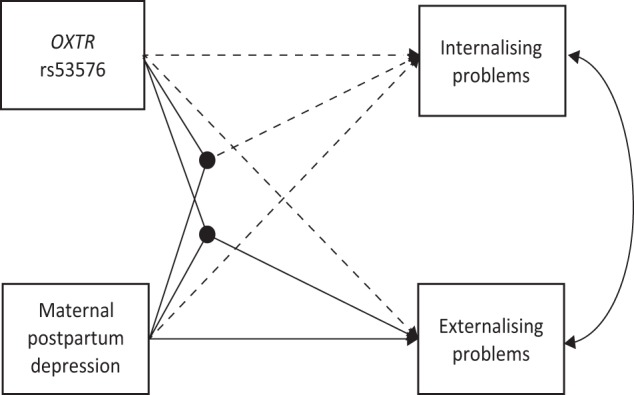
Structural equation model for the interaction effect of oxytocin receptor gene (*OXTR*) rs53576 genotype × maternal postpartum depression on child behavioural problems. Solid lines indicate statistically significant paths (i.e., 95% confidence interval does not cross zero), while dashed line indicate non-significant paths. Covariates (child gender, gestational age, birth weight, maternal education, house income, and history of maternal affective disorder) were adjusted for in this model.

**Figure 2:**
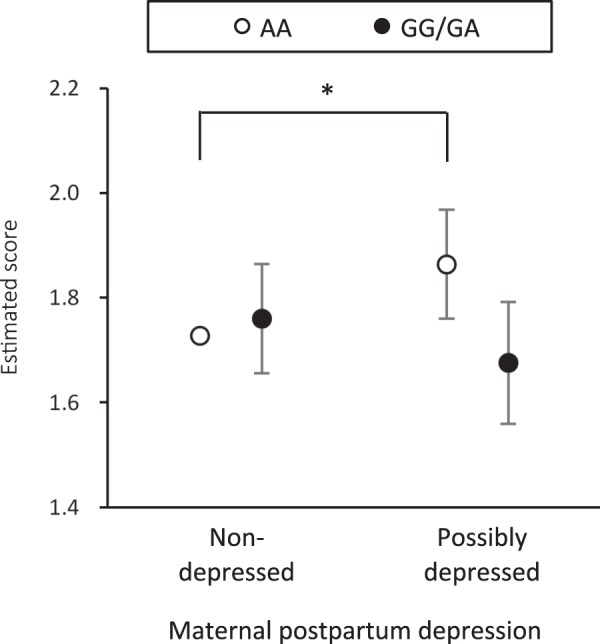
Interaction effect of oxytocin receptor gene (*OXTR*) rs53576 genotype × maternal postpartum depression on externalising problems. Scores are standardized values derived from the structural equation model. Covariates (child gender, gestational age, birth weight, maternal education, house income, and history of maternal affective disorder) were adjusted for. Error bars indicate 95% confidence interval (CI). *The 95% CI does not cross the estimated score for the baseline group (AA genotype offspring with non-depressed mother), which is equivalent to *p* < 0.05.

Table 1 and Fig. 1 present the results of SEM analysis. In the crude model, there was a significant interaction effect of *OXTR* × maternal postpartum depression on externalising problems (*β* = −0.210, 95% CI −0.359 to −0.062). Such an interaction of *OXTR* × maternal postpartum depression remained significant when several covariates were adjusted for: *β* = −0.185, 95% CI −0.330 to −0.040 for the adjusted final model. To examine the interaction effect, we calculated the difference in the coefficients among the four groups; that is, reparameterization was made for the adjusted final model (see Table 2). The association between maternal postpartum depression and externalising problems in offspring was evident in children with the AA genotype (*β* = 0.136, 95% CI 0.032 to 0.240), but not in those with GG/GA genotypes (Fig. 2). As for internalising problems, there was no interaction effect of *OXTR* × maternal postpartum depression in either the crude or adjusted model. Then, we examined any sex differences in the interaction effect by applying multiple group analysis in the framework of SEM. We found no significant difference in the interaction effect of *OXTR* × maternal postpartum depression on externalising problems: *β* = −2.301, 95% CI −6.841 to 2.239, *p* > 0.05.

**Table 1:** Structural equation model for the interacting effect of oxytocin receptor (*OXTR*) rs53576 genotype × maternal postpartum depression on child behavioural problems.

	Internalising problems		Externalising problems	
	*β*	95% CI	*β*	95% CI
**Crude model**				
*OXTR* ^a^	−0.072	−0.162 to 0.018	0.034	−0.056 to 0.125
Maternal postpartum depression^b^	0.155	0.001 to 0.309*	0.184	0.055 to 0.314*
OXTR^a^ × Maternal postpartum depression^b^	−0.023	−0.172 to 0.126	−0.210	−0.359 to −0.062*
**Adjusted model** ^**c**^				
*OXTR* ^a^	−0.067	−0.157 to 0.022	0.036	−0.053 to 0.125
Maternal postpartum depression^b^	0.119	−0.034 to 0.271	0.177	0.043 to 0.312*
*OXTR*^a^ × Maternal postpartum depression^b^	−0.025	−0.166 to 0.115	−0.185	−0.330 to −0.040*

*β*’s correspond to standardized coefficients.

^a^AA was coded as 0 and GG/GA as 1.

^b^Non-depressed mothers were coded as 0 and possibly depressed mother as 1.

^c^Covariates included child gender, gestational age, birth weight, maternal education, house income, and history of maternal affective disorder.

*The 95% confidence interval (CI) does not cross zero, which is equivalent to *p* < 0.05.

**Table 2:** Difference of the score of child behavioural problems in each group compared with baseline group (AA genotype offspring with non-depressed mother) in relation to oxytocin receptor (*OXTR*) rs53576 genotype (AA vs. GG/GA) and maternal postpartum depression. Covariates (child gender, gestational age, birth weight, maternal education, house income, and history of maternal affective disorder) were adjusted for.

	Internalising problems		Externalising problems	
	*β*	95% CI	*β*	95% CI
AA genotype offspring with non-depressed mother (*baseline*)	1.773	—	1.728	—
AA genotype offspring with possibly depressed mother	0.090	−0.024 to 0.205	0.136	0.032 to 0.240*
GG/GA genotype offspring with non-depressed mother	−0.135	−0.235 to −0.035*	0.032	−0.072 to 0.137
GG/GA genotype offspring with possibly depressed mother	0.000	−0.097 to 0.096	−0.052	−0.168 to 0.064

*β*’s correspond to standardized coefficients.

^*^The 95% confidence interval (CI) does not cross zero, which is equivalent to *p* < 0.05.

Although independent main effects of the risk factors (*OXTR* and maternal postpartum depression) were not included in our hypothesis, it is of interest to note that the main effect of maternal postpartum depression in both the crude and adjusted models was significant (*β* = 0.184, 95% CI 0.055 to 0.314 in the crude model; *β* = 0.177, 95% CI 0.043 to 0.312 in the adjusted model) for externalising problems, indicating that offspring whose mother was afflicted with postpartum depression had higher levels of externalising problems than those whose mothers did not experience postpartum depression (Table 1). In addition, the similar risk-increasing effect of maternal postpartum depression, as a main effect, was present for internalising problems in the crude model (*β* = 0.155, 95% CI 0.001 to 0.309); however, the effect became non-significant when covariates were adjusted for (*β* = 0.119, 95% CI −0.034 to 0.271) (Table 1).

## Discussion

To our knowledge, this is the first study to demonstrate the interaction effect of *OXTR* rs53576 genotype × environmental hazard on mental health in children. We found a significant interaction effect of *OXTR* genotype and maternal postpartum depression on externalising problems in children. As hypothesized, the effect of maternal postpartum depression was found for the A allele of *OXTR* rs53576 genotype, but not for the G allele. The finding of time-sequential relation, and not cross-sectional relation between maternal postpartum depression and *OXTR* rs53576 genotype that occurs early in life (i.e. in the neonate) and subsequent behavioural problems in offspring (6 years of age) alludes to the presence of a causal link. Further, the current findings are generalizable since the sample examined was representative of the general population and the attrition rate was small in our birth cohort despite the fact that longitudinal studies, in general, encounter drawbacks due to high drop-out rates. Moreover, we applied parallel-processing analysis for two outcome measures (internalising and externalising problems), which allowed for covariance between the outcomes. In fact, the covariance was significant in the adjusted final model: *β* = 0.316, 95% CI 0.236 to 0.397. Thus, this parallel-processing approach precluded commonly-occurring false positive findings due to multiple separate testing.

The association of maternal postpartum depression with increased externalising problems was shown only in children with the AA genotype, as we *a priori* hypothesized, not in those with GG/GA genotypes. This is in line with previous study^22^ showing that the A allele, but not the G allele, genotype of *OXTR* rs53576 is associated with aggressive behaviours in adolescence when individuals are exposed to high life stress as a harmful environmental factor. In this previous study, stressful life events included a wide range of negative events including serious illness in family members. Maternal postpartum depression is not only a psychological problem, but also involves physiological conditions. Therefore, the present finding provides additional evidence supporting the role of the A allele of *OXTR* rs53576 as a vulnerability marker for developing behavioural problems by operating together with environmental burdens, i.e., maternal postpartum depression. Previous animal studies have indicated that oxytocin interacts with the dopamine reward system, serotonin system, and immune system (reviewed in^4,35^). Recent studies suggest that through this endogenous oxytocin system, early environmental factors affect the resilience to stress^35^, and balance of preference between novelty and stability^36^. Low resilience to stress and high novelty seeking are common characteristics in children with externalising problems^37,38^. One of possible mechanisms to explain the present finding is: the interaction between A allele of *OXTR* rs53576 and maternal postpartum depression may dysregulate the oxytocin system, which in turn decreases the resilience to stress, and increases novelty seeking, leading to the emergence of externalising problems.

Furthermore, it is noteworthy that the interaction effect of *OXTR* rs53576 genotype × maternal postpartum depression on externalising problems in the present study was evident even when the history of maternal affective disorder was adjusted for. On the basis of this finding, one may speculate that environmentally-mediated effects (i.e. postnatal depression) rather than genetic predisposition are likely to underlie the mechanism in the present finding. However, this view remains tentative since history of mental disorders is merely a proxy. To clarify genetic confounding effects in the present study, further studies with advanced genetic investigations (e.g. genome analysis of both mother and child) are required. Another plausible interpretation is disturbed mother-child relationships. There has been a suggestion that maternal postpartum depression may disrupt the normal mother-child relationship, thereby impairing child development and mental health^39^. Thus, it is plausible that maternal postpartum depression may hamper positive communication between the mother and child during infancy, and the resultant impaired relationship could predispose the child to externalising problems wherein the A allele of *OXTR* rs53576 genotype may intervene.

As mentioned above, the gene × environment interaction effect on mental health has been explained by several models such as the diathesis-stress model (genetic variance associated with a stressful environment) and the susceptibility model (genetic variance related to both stressful and beneficial environments). Superiority of either model with regard to appropriateness to account for the gene × environment interaction effect has been debated^40,41^. In the present study, the environmental factor we considered was maternal postpartum depression, which can be viewed as a stressful environment rather than a supportive environment. However, the issue on evaluating the hypothesised models is outside the scope of the present study. Future studies are clearly needed to tackle the gene × environment interaction effects, and to elucidate the role of stressful as well as supportive environments in the effects.

Regarding internalising problems as another primary outcome measure in the present study, we found no interaction effect of *OXTR* rs53576 genotype × maternal postpartum depression, which is contradictory to our anticipated results. This result is incompatible with that of a previous study showing an interaction effect of *OXTR* rs53576 genotype (especially the A allele) and history of maternal depression in the first 5 years of a child’s life on depressive symptoms in offspring at age 15 years^18^. One possible explanation for these inconsistent findings is the age at which child mental health was assessed. In the present study, internalising problems were measured at 6 years of age, while in the previous study^18^, depressive symptoms were evaluated at 15 years. In contrast to externalising problems, internalising problems tend to become conspicuous with increasing age, due to the association between cognitive development and emergence of internalising problems^42^. Therefore, the effect of the interaction of *OXTR* rs53576 genotype × maternal depression on internalising problems in offspring may become clearer at later developmental stages. Another interpretation is varying grouping of *OXTR* rs53576 genotype in different studies. In the present study, the GG and GA genotypes were combined, while in the previous study, AA and GA genotypes were conflated^18^. In general, in research based on Asian samples, the GG and GA genotypes have been amalgamated due to a low frequency of the GG genotype in those populations (e.g.^22,34^), whereas a low frequency of the AA genotype in European or American samples has led to integration of the AA and GA genotypes (e.g.^8,20^). However, as mentioned above, there is evidence indicating that the GG and GA genotypes share common characteristics such as psychological properties^19^ and personality traits^13^. Hence, these factors theoretically underpin our choice of combining the GG and GA genotypes.

In the present study, we used all data available in the SEM approach. This was achieved under the assumption of missing at random (MAR). If MAR was not held, the estimations obtained would be somehow biased. To clarify any influence of missingness (i.e., any presence of not missing at random, NMAR) on the present findings, we repeated the same SEM procedure by constraining the sample of participants who had complete data on both principal variables (i.e., maternal postpartum depression and behavioural problems) (i.e., ‘listwise deletion’ sample, *n* = 415). The coefficient for the interaction effect of *OXTR* rs53576 × maternal postpartum depression on externalising problems in the listwise-deletion sample remained virtually unchanged and significant (*β* = −0.173, 95% CI −0.305 to −0.042), suggesting that the MAR assumption for the SEM analysis was not violated. Regarding internalising problems, no significant interaction effect of *OXTR* rs53576 × maternal postpartum depression in the listwise-deletion sample remained the same (*β* = −0.019, 95% CI −0.152 to 0.113).

The present study has several strengths. First, the present study was predicated on a longitudinal birth cohort, which comprises a representative sample of the general population. Although there were a few differences in sociodemographic variables between the final sample group employed in this study and the remaining samples in our birth cohort as discussed below (see *Supplement*, Table 1s), principle variables (i.e., maternal postpartum depression and child behavioural problems) were almost identical between the two samples. Second, we used parallel-processing SEM analysis to handle two outcome measures, internalising and externalising problems, that are highly correlated with each other. If testing was done separately for each outcome, shared underlying characteristics, but not necessarily unique nature of the two outcomes, may have contributed to false positive findings.

The present study also has several limitations. First, the final sample examined had a lower probability of preterm birth, a higher proportion of maternal education background and a higher probability of maternal affective disorders compared with the remaining samples in our birth cohort (Table 1s). If these factors were not adequately handled, the results may have been biased. In the analyses, these factors were modelled as covariates—any confounding effects related to these variables that could be eliminated. However, there may be a disproportionate distribution in unobserved variables between the samples and thus these were unable to be adjusted for in the present study, which may have led to bias. Second, child mental health was assessed based on the parental report, and thus, measurement error cannot be ruled out. Although parental reports on child behavioural problems, both on internalising and externalising problems, show high agreement with diagnoses by clinicians^43^, future studies need to measure mental health using objective methods such as a professional ascertainment with direct interviews and observation. Third, we did not include a factor of maternal mental health, especially maternal depression, when children were 6 years old as a covariate in the analysis. It is possible that maternal depression at the time of reporting of child behaviour may have influenced maternal reports and biased the findings. This further indicates a need to measure child behavioural problems using objective methods. Fourth, the present study analysed only one single SNP, rs53576. Although rs53576 is the most well-investigated *OXTR* genotype, there are also other SNPs of the *OXTR* gene associated with child mental health (e.g., rs2254298^44^). Further studies using various SNPs for the *OXTR* gene are needed.

In summary, the present study indicates an interaction effect of oxytocin receptor genotype (the A allele of *OXTR* rs53576) × environmental factors in early life (maternal postpartum depression) on child behavioural (externalising) problems. Our findings add to the evidence that the A allele of *OXTR* rs53576 genotype is one of the vulnerability biomarkers for predisposition to mental problems in children, in cooperation with environmental burdens such as maternal postpartum depression. For understanding mechanisms of this gene × environment effect, further studies are needed.

## Methods

### Participants

This study was conducted as part of an ongoing cohort study in Japan, the Hamamatsu Birth Cohort Study for Mothers and Children (HBC Study). Participants included mothers (*n* = 1138) and their infants (*n* = 1258) born between 2007 and 2012. The participating children did not show any specific departure from the national statistics of demographic and perinatal data in Japan^45^, suggesting that this cohort is representative of the general population. Furthermore, the drop-out rates of our cohort were very low (<10% at 2-year follow-up after birth)^45^. The study protocol was approved by the Hamamatsu University School of Medicine and University Hospital Ethics Committee and performed in accordance with the Declaration of Helsinki. Written informed consent was obtained from each mother for her own and her infant’s participation.

In the present study, children who met the following criteria were excluded: First, we excluded children who died before one year (*n* = 4) or had birth weights below 1000 g (*n* = 1). Second, due to financial constraints, we randomly selected 600 children for analysis for DNA specimens; however, 2 participants among them were identified as having Down syndrome and were excluded; thus, the DNA samples were analysed from 598 offspring. Third, we eliminated children for whom we failed to analyse *OXTR* rs53576 genotype (*n* = 30). This resulted in a sample of 568 children (277 female, 291 male). Characteristics of the final samples employed in the present study and the remaining samples from the HBC study are shown in the *Supplement*, Table 1s.

### Measures for behavioural problems in children

Child internalising and externalising problems were measured using the Strength and Difficulties Questionnaire (SDQ)^46^ by parental report when children were 6 years old. The SDQ consisted of 28 items with five domains: conduct problems, hyperactivity-inattention, emotional symptoms, peer problems, and prosocial behaviour. Parents answered items based on the child’s behaviour over the last six months. Each item was scored on a 3-point scale in which 0 equated to “not true,” 1 “somewhat true,” and 2 “certainly true.” Using a factor analytic approach, previous studies have shown that internalising problems are defined as combining the emotional symptoms and peer problems domains and externalising problems are defined as combining the two domains of conduct problems and hyperactivity-inattention^47,48^. Because the prosocial behaviour domain is considered to be a factor independent from internalising and externalising problems^47,48^, this domain was not used in the present study.

### Maternal postpartum depression

Maternal postpartum depression was measured using the Edinburgh Postnatal Depression Scale (EPDS)^49^ when children were 2 months and 10 months old. The EPDS consisted of 10 items. Mothers responded to each of the items based on how they felt in the past 7 days. Each item was scored on a 4-point scale in which 0 = “No, not at all,” 1 = “No, not very often,” 2 = “Yes, most of the time,” and 3 = ”Yes, all the time.” In Japan, the recommended score to distinguish depression on the EPDS is 8/9 points^50–52^, which is lower than that in the West (12/13)^49^. This cut-off difference has been accounted for by the documented tendency for Japanese women to be less expressive of their feelings than Western women^53^ and, hence, reports from Japan have followed this lenient cut-off^50–52^. Thus, in the present study, mothers were rated as “possibly depressed” and “non-depressed” based on an EPDS score ≥ 9 or <9, respectively, at either 2 months postpartum or 10 months postpartum; we opted to use a relatively wide window for the risk period, based on previous reports^31^.

### *OXTR* rs53576 genotype analysis

DNA samples were collected from children aged 32 months, 40 months, 4.5 years, or 6 years (corresponding to the follow-up assessment sessions) by means of a buccal swab (Isohelix SK-2S, Harrietsham, UK). DNA isolation was conducted (Isohelix DDK-50), and the extracted DNA samples were stored at −20 °C.

Genotyping was conducted using the Japonica array (Toshiba, Tokyo, Japan), which is an SNP typing array designed for Japanese populations^54^. The quality of isolated DNA was tested, and the samples that did not meet the quality criteria (A260/280 ratio < 1.6 or DNA fragment < 10 kbp) were excluded from further analysis (n = 29). Amplification was performed using the AxiomTM Reagent Kit (Thermo Fisher Scientific Inc, Waltham, MA, USA), and then genotyping was carried out using AxiomTM Analysis Suite 1.1.1.66 (Thermo Fisher Scientific Inc, Waltham, MA, USA). One sample (*n* = 1) was removed because it was not identified for the rs53576 genotype.

### Covariates

Child gender, gestational age, birth weight, maternal education, household income, and history of maternal affective disorder were included as covariates. Child gestational age was dichotomized into non-preterm birth (≥37 weeks) and preterm birth (<37 weeks). Birth weight was categorized into non-low birth weight (≥2500 g) and low birth weight (<2500 g). Maternal education was categorized into three levels: low (<12 years), middle (12 to 15 years), and high (>=16 years). Household income was reported during pregnancy and categorized into three levels: low (<annual 3 million JPY), middle (annual 3 to 8 million JPY), and high (≥annual 8 million JPY). History of maternal affective disorder was assessed during pregnancy using the DSM-IV-TR by trained clinicians. If mothers reported a history of major depressive disorder or bipolar disorder, they were categorized into subjects with “affective disorder” and the remaining mothers as non-affective disorder.

### Statistical analysis

Given that patients with GG and GA genotypes share common psychological characteristics (e.g.^13,19^), *OXTR* rs53576 genotype was dichotomized into GG/GA and AA. To examine any interaction effect of *OXTR* rs53576 genotype and maternal postpartum depression on internalising and externalising problems in offspring, parallel-processing structural equation modelling (SEM) was employed throughout the estimation. This parallel process allows for covariance between our main outcome measures (internalising and externalising problems) and thus, averts multiple testing and minimizes type I error. Since the scores of SDQ showed non-normal distribution (Shapiro-Wilk Test, all *p* < 0.001 for both measures), we employed maximum likelihood to derive the estimation of parameters and utilized bootstrapping procedures (*n* = 10,000) to yield non-symmetrical confidence intervals (CI)^55^. Internalising and externalising problems were set as the outcome variables, whereas *OXTR*, maternal postpartum depression, and the interaction effect of *OXTR* × maternal postpartum depression were entered as predictor variables. First, we conducted SEM analysis without covariates, and then we incorporated covariates into the model. Because some offspring were born from the same mother (siblings: n = 30 pairs; twins = 9 pairs), such clustering (i.e., family clustering) was allowed for in the analyses using a Huber sandwich estimator. Statistical analysis was performed using Mplus Version 8 (Muthén and Muthén, 2017). If the range of the 2.5 and 97.5 percentile CIs did not cover the null value (i.e., 0), it was judged to correspond to *p* < 0.05. In these SEM analyses, we utilized all data available using the full information maximum likelihood algorithm.

## Supplementary information

Table 1s

## Data Availability

The datasets generated during and/or analysed during the current study are available from the corresponding author.
