# Supplementary material for: Interaction effect of oxytocin receptor (OXTR) rs53576 genotype and maternal postpartum depression on child behavioural problems
Source: Sci Rep. 2019 May 22;9:7685. doi: 10.1038/s41598-019-44175-6 (PMC6531431; doi:10.1038/s41598-019-44175-6)
Supplement: Supplementary file 1 — Table 1s [file 41598_2019_44175_MOESM1_ESM.docx]

**Supplementary information**

**Interaction effect of oxytocin receptor (OXTR) rs53576 genotype and maternal postpartum depression on child behavioural problems**

Damee Choi^1^, Kenji J Tsuchiya^1^, Nori Takei^1,2*^

^1^Research Centre for Child Mental Development, Hamamatsu University School of Medicine, Hamamatsu, Japan

^2^Institute of Psychiatry, King’s College London, London, UK

**Table 1s.** Characteristics in the final sample analyzed in the present study and the remaining sample from the HBC study

|  | Final sample | Sample not included in the present study^a^ | Group difference (*p* value) |
| --- | --- | --- | --- |
| Child gender, n (%) |  |  | 0.833^b^ |
| Girl | 277 (48.8%) | 329 (48.2%) |  |
| Boy | 291 (51.2%) | 354 (51.8%) |  |
| Child gestational age, n (%) |  |  | 0.048^b^ |
| ≥ 37 weeks (non-preterm birth) | 539 (94.9%) | 629 (92.1%) |  |
| < 37 weeks (preterm birth) | 29 (5.1%) | 54 (7.9%) |  |
| Child birth weight, n (%) |  |  | 0.123^b^ |
| ≥ 2500 g (non-low birth weight) | 504 (88.7%) | 586 (85.8%) |  |
| < 2500 g (low birth weight) | 64 (11.3%) | 97 (14.2%) |  |
| Maternal education, n (%) |  |  | 0.044^b^ |
| < 12 years | 243 (4.1%) | 49 (7.1%) |  |
| 12 to 15 years | 386 (69.7%) | 474 (69.4%) |  |
| >=16 years | 149 (26.2%) | 160 (23.4%) |  |
| Annual household income, n (%) |  |  | 0.059^b^ |
| <3 million JPY | 25 (4.4%) | 50 (7.3%) |  |
| 3 to 8 million JPY | 439 (77.3%) | 497 (72.8%) |  |
| ≥ 8 million JPY | 104 (18.3%) | 136 (19.9%) |  |
| History of maternal affective disorder, n (%) |  |  | 0.009^b^ |
| Non-affective disorder | 499 (87.9%) | 630 (92.2%) |  |
| Affective disorder | 69 (12.1%) | 53 (7.8%) |  |
| Maternal postpartum depression, n (%) |  |  | 0.545^b^ |
| Non-depressed mothers | 394 (91.0%) | 314 (89.7%) |  |
| Possibly depressed mothers | 39 (9.0%) | 36 (10.3%) |  |
| Child behavioural problems |  |  |  |
| Internalising problems |  |  | 0.822^c^ |
| Mean (SD) | 4.03 (2.77) | 4.01 (2.79) |  |
| Median (IQR) | 4 (2 to 5) | 3 (2 to 6) |  |
| Range | 0 to 13 | 0 to 14 |  |
| Externalising problems |  |  | 0.562^c^ |
| Mean (SD) | 5.74 (3.37) | 5.86 (3.39) |  |
| Median (IQR) | 5 (3 to 8) | 6 (3 to 8) |  |
| Range | 0 to 19 | 0 to 15 |  |

a. Children who had died before one year (*n*=4), those diagnosed with Down syndrome (*n*=2) or those whose birth weight had been under 1000 g (*n*=1) were excluded.

b. Chi-squared test.

c. Mann-Whitney test.
